# Resurrected ‘ancient’ *Daphnia* genotypes show reduced thermal stress tolerance compared to modern descendants

**DOI:** 10.1098/rsos.172193

**Published:** 2018-03-21

**Authors:** Aime'e M. Yousey, Priyanka Roy Chowdhury, Nicole Biddinger, Jennifer H. Shaw, Punidan D. Jeyasingh, Lawrence J. Weider

**Affiliations:** 1 Department of Biology, University of Oklahoma, 730 Van Vleet Oval, Norman, OK 73019, USA; 2 Program in Ecology and Evolutionary Biology, University of Oklahoma, 730 Van Vleet Oval, Norman, OK 73019, USA; 3 Department of Integrative Biology, Oklahoma State University, 501 Life Sciences West, Stillwater, OK 74078, USA

**Keywords:** local adaptation, resurrection ecology, climate change

## Abstract

Understanding how populations adapt to rising temperatures has been a challenge in ecology. Research often evaluates multiple populations to test whether local adaptation to temperature regimes is occurring. Space-for-time substitutions are common, as temporal constraints limit our ability to observe evolutionary responses. We employed a resurrection ecology approach to understand how thermal tolerance has changed in a *Daphnia pulicaria* population over time. Temperatures experienced by the oldest genotypes were considerably lower than the youngest. We hypothesized clones were adapted to the thermal regimes of their respective time periods. We performed two thermal shock experiments that varied in length of heat exposure. Overall trends revealed that younger genotypes exhibited higher thermal tolerance than older genotypes; heat shock protein (hsp70) expression increased with temperature and varied among genotypes, but not across time periods. Our results indicate temperature may have been a selective factor on this population, although the observed responses may be a function of multifarious selection. Prior work found striking changes in population genetic structure, and in other traits that were strongly correlated with anthropogenic changes. Resurrection ecology approaches should help our understanding of interactive effects of anthropogenic alterations to temperature and other stressors on the evolutionary fate of natural populations.

## 1. Introduction

Over the past century, the mean global temperature has increased by nearly 1°C and is predicted to increase another 1.5°C over the course of the next century [1]. Species that inhabit terrestrial, aquatic and marine systems have responded to such changes in a variety of ways, ranging from alterations in distributional ranges to shifts in phenology through modifications of physiology and behaviour, among other responses over time [1,2]. However, due to the rapid pace of predicted temperature increases, concerns of how quickly a given species may adapt have become a focus of recent climate change studies [3]. Understanding how, or if, a species can adapt to future climate change scenarios is of critical importance [2], as such changes will impact demography, and interactions across trophic levels [4–7]. Owing to shifts in phenology, novel interactions across trophic levels are possible and have been observed in a variety of ecosystems [2,5]. The persistence of such interactions could lead to unpredictable outcomes. Moreover, as species continue to adapt to the changing climate, the aforementioned effects have the potential to occur at such a rapid rate, that organisms will not be able to adapt quickly enough, thus, eliciting multiple negative consequences, including extirpation or extinction [4]; IPCC, 81 2014 [8].

Much previous work (e.g. [2,3]) has focused on terrestrial systems, where responses such as changes in species ranges or vegetational shifts (e.g. [2]) have been of particular interest [1]. Much less attention has been focused on freshwater ecosystems, such as lakes, which are also sensitive to warming and the changing global climate [5,9]. Rising temperatures have major impacts on the functioning of aquatic ecosystems [10]. Moreover, temperature is a powerful selective force affecting the ecology and evolutionary potential of organisms [11]. Such evolutionary shifts can thus have far-reaching impacts. Indeed, thermal tolerance at the individual level has been linked to effects noted in the community, food web and ecosystem as a whole [2,5].

*Daphnia* is a genus of freshwater zooplankton (Cladocera, Anomopoda), and is negatively affected by rising temperatures, which includes impacts such as aberrant ageing effects or reduced lifespan [12–14]. Daphniids are well known for their adaptive responses to environmental changes [15,16]. Therefore, they may possess the potential to rapidly evolve in response to rising temperatures. Understanding the evolutionary responses of populations of *Daphnia* to changes in temperature is paramount because of their status as a keystone species. Daphniids are not only involved in interactions with both primary consumers and secondary predators, but they are also critical regulators of algal biomass and serve as prey for invertebrate and vertebrate predators [16]. Thus, disruption to the health of *Daphnia* populations in lake ecosystems potentially leads to detrimental consequences across multiple trophic levels [16–18].

Rising temperatures can lead to microevolutionary responses in populations of *Daphnia* that can shift a variety of traits including phenology [18], physiology (e.g. respiration, haemoglobin production [19]); among other physiological traits [7,16,18,20], behaviour [19,21], life history [10,22] as well as thermal tolerance [23–25]. Previous work has documented increased levels of heat shock protein production in *Daphnia* as a response to biotic (i.e. predation [26]) and abiotic (i.e. temperature [27]) stressors. These stressors have the potential for causing significant impacts (i.e. cellular damage) on organisms [13,28]. Although rising temperatures alone are unlikely to result in total extirpation of *Daphnia* from their habitat (although see Coutant & Brook [29], for a review of thermal pollution effects on aquatic organisms), thermal stress has been experimentally demonstrated to affect the genus metabolically [21,30] and physiologically [25,31]. Nevertheless, comparatively little is known about how rising temperatures have shaped thermal physiology in natural *Daphnia* populations (e.g. [25]), with some exceptions, especially when coupled with other environmental stressors, such as pollutants [32] or invasive species [33].

In this study, we used methods in ‘resurrection ecology’ [34,35] to investigate the effect of temperature increase on four subpopulations of *Daphnia pulicaria* over time. Specifically, we took advantage of the reproductive mode of *Daphnia*, termed cyclical parthenogenesis, in which there is an asexual phase (i.e. apomictic cloning), and a sexual phase, the result of which produces eggs that are encased in a resistant scleroterized structure known as an ephippium. Ephippia are produced under stressful environmental conditions and released into the water column [16], where some will settle to the bottom (benthic) sediments until environmental cues stimulate hatching. Some ephippia, however, never hatch and are buried in the sediments over time. These ephippia can be found in deep sediment layers extracted from the benthic sediments, with some layers dating back centuries [36,37]. These dormant propagules are stimulated to hatch in the laboratory (i.e. ‘resurrection ecology’ [34,35]) and represent natural *Daphnia* subpopulations from past environments that are then available for experimentation (e.g. [37]). This approach allows one to track the evolutionary trajectories of historical populations (akin to uncovering ‘physiological fossils') in response to environmental variables. Resurrection ecology is useful in climate change research for the development of predictive models that potentially enable the characterization of organismal (in our case, *Daphnia*) responses to future climate change conditions [36].

Most *Daphnia* studies to date have examined adaptation on a shorter time scale ranging from generations to seasons [38–40]. Our unique system expands the temporal dimension, thereby enabling the study of adaptation over years to centuries. The clones used herein exhibited striking microevolutionary dynamics to changing nutrient conditions in South Center (SC) Lake (MN) over a centennial–millennial time scale [37]. Specifically, Frisch *et al*. [37] determined that *Daphnia* allelic and genotypic frequencies responded to increased phosphorus supply. Moreover, Roy Chowdhury *et al*. [41] demonstrated that eutrophication resulted in genetic changes as detected by shifts in physiology and gene expression patterns in this same system. As an extension to these studies, we propose that rising temperatures may play a role as a selective force on the subpopulations of *Daphnia* from this lake (see Material and methods section for more details). The impacts of rising temperatures on this organism are a cause for concern, as it may impair the microevolutionary potential of *Daphnia* to respond to elevated temperatures in an adaptive manner [10,22,25,42] (A.M. Yousey and L.J. Weider, personal observation). Previous work has investigated thermal adaptation over a broader time scale from birth to the release of the third clutch and produced evidence for plasticity rather than local adaptation (A.M. Yousey and L.J. Weider, personal observation). This study aims to further explore thermal adaptation through an assessment of different traits (i.e. survivorship and expression of a heat shock protein gene).

We specifically addressed the following questions: (i) Do *Daphnia* exhibit any temporal (time-specific) shifts in thermal tolerance across a centennial-level time scale? (ii) Among *Daphnia* temporal subpopulations, is there evidence of local adaptation to the thermal environments of their respective time periods? If so, (iii) can we assess this adaptive response by measuring differential survivorship among clones, when subjected to a thermal stress? Finally, (iv) does variation in the expression of heat shock proteins underlie variation in heat tolerance?

## 2. Material and methods

### 2.1. Sample collection

The clones for our study were hatched from ephippia isolated from a sediment core harvested from a eutrophic lake in Minnesota, South Center (SC) Lake, in July 2010. Clonal lines were established in the laboratory from hatchlings obtained from separate sediment layers in the core (see below). See Frisch *et al*. [37] regarding protocols to obtain cores including dating sediment layers using ^210^Pb and ^137^Cs radio-isotopes, and hatching methodologies. A total of 10 genetically distinct clones isolated from an SC Lake sediment core collected in summer 2010 were used in the experiments outlined below. Nine of these clones were examined by Frisch *et al*. [37] and are known to exhibit physiologically distinct phenotypes for carbon : phosphorus (C : P) processing. These same nine clones and a 10th clone were used in a life-history experiment that examined response to various temperatures (A.M. Yousey and L.J. Weider, personal observation). All animals were collected from the same core, hatched concurrently and maintained under identical conditions. Experimental clones were isolated from the 4–8, 20–24, 52–56 and 60–64 cm layers of the core. See Morton *et al*. [43] for details about hatching success for the deepest layer.

Layers are referenced hereafter with the following classifications, which match the nomenclature used in previous studies in this system [37,41,43]: clones from the 4–8 cm layer are referred to as ‘modern’ (M), 20–24 cm as ‘recent’ (R) and 52–56 and 60–64 cm as ‘ancient’ (A). Modern clones are representatives of present-day ambient summer *ca* AD 2002–2008, when air temperatures of SC Lake region averaged 25°C [44,45]. Recent clones existed during a cooling period roughly 40–50 years ago, *ca* AD 1967–1977, with air temperatures averaging 20°C [45]. Previous work [37] had isolated two ancient clones from the 52–56 cm layer, estimated to have lived AD 1536–1646, and another two from the 60–64 cm layer of SC, estimated to have lived AD 1301–1418. These ancient clones pre-date the 1800s and thus, were deposited in lake sediments during the Little Ice Age (*ca* 1300–1800), when summer ambient air temperatures in the northern hemisphere are estimated to have been approximately 1–10°C lower than present-day temperatures [46]. Thus, we assume that each group of clones has adapted to different temperature regimes, and that their thermal tolerances will reflect these different thermal histories.

### 2.2. Experimental design

Two separate acute thermal shock experiments were conducted. The first experiment was conducted in summer 2012 and lasted for approximately 3 h. The second experiment was conducted in spring 2015 and lasted approximately 72 h. The purpose of these two experiments was to explore the differences in thermal tolerance of clones between the three time periods and determine the robustness of these patterns after clones were exposed to increasing temperatures under different (i.e. time) exposure conditions. (Note: experiment 1 was conducted at Oklahoma State University, while experiment 2 was conducted at the University of Oklahoma.)

### 2.3. Experiment 1: short-term acute thermal shock

To test the hypothesis that *Daphnia* are locally adapted to their respective thermal regimes, nine clones across three time periods were employed for experimentation in this study (the same nine clones used in the Frisch *et al*. [37] study): three modern clones (M1, M2 and M3), three recent clones (R1, R2 and R3) and three ancient clones (A1, A2 and A4). Clones were sequentially exposed to the following temperature treatments for 30 min periods: 28°C, 32°C, 34°C, 35°C and 36°C. We predicted clones that were locally adapted to the highest summer average temperatures would elicit the highest survivorship throughout the highest thermal exposure. More specifically, modern clones would exhibit the greatest rates of survivorship with exposure to increasing temperatures up to 36°C, while recent clones would have lower rates of survivorship, and ancient clones even lower survivorship.

Animals were raised for two generations at 20°C prior to experimentation to reduce maternal effects [47]. A grand-maternal generation was established with animals obtained from stock cultures acclimated to laboratory conditions at 20°C. Third-clutch individuals were used to establish maternal and experimental animal generations, as the third clutch is typically healthier and more robust, relative to neonates from either the first or second clutches [48].

Experimentation followed the methods described in Yorks *et al*. [49]. For each clone, there were three replicate 60 ml jars containing 50 ml of artificial pond water (COMBO; [50]) and five neonates per jar. A total of 27 experimental vessels were established that were submitted to the same increasing temperature series, as outlined above. Background and handling mortality rates were estimated in a control jar at 20°C for each clone (i.e. five neonates per jar). *Daphnia* were fed 1 mgC l^−1^ high phosphorus (HiP) *Scenedesmus acutus* algae every day as high concentration food minimizes food stress and reduces negative effects that could impact survivorship. Seven-day-old *Daphnia* were used in our experiments, and had been maintained under ambient (20°C) temperatures prior to experimentation. We chose this age-class in order to minimize thermal stress effects that have been observed for younger (juvenile) animals (L.J.W., personal observations), and we wanted to minimize the possibility of animals reaching reproductive age prior to the conclusion of our experiments.

### 2.4. Quantification of heat shock protein (hsp70) gene expression

The gene expression of heat shock protein (hsp70) was quantified following exposure to room temperature, 20°C, and two of our temperature treatments, 32°C and 36°C. We collected 10 gravid females from each clone and monitored for the release of neonates every 12 h. Neonates were raised individually and fed HiP at 3 mgC l^−1^ for 5 days at room temperature (i.e. 20°C). Five-day-old neonates underwent temperature shock treatments by placing them in pre-heated 50 ml jars in a water bath at the three temperatures. After 30 min of incubation, 10 neonates per clone per temperature treatment were transferred to a 1.5 ml microcentrifuge tube. Any remaining water was carefully removed by pipetting. Afterwards, 500 µl of Trizol® (Invitrogen) was added to the tube and *Daphnia* were crushed by hand for 5 min using a plastic pestel. Another 500 µl of Trizol was added and tubes were immediately stored at −80°C for downstream analyses. Each of the tested clones were exposed to these three temperatures separately. Each temperature/clone combination was replicated five times.

To each 1 ml of Trizol, 200 µl of chloroform was added to frozen samples at room temperature. Samples were centrifuged at 11 600 rcf for 15 min at 4°C. The upper aqueous phase containing the RNA was transferred to a new RNAse-free 1.5 ml microcentrifuge tube. RNA was precipitated by adding 250 µl of 100% ethanol at room temperature. RNA purification and DNAse treatment (to rid any potential contaminating genomic DNA) were conducted using RNeasy mini-spin kit (Qiagen; Hilden, Germany) following the manufacturer's instructions. RNA quality was determined using a NanoDrop spectrophotometer (NanoDrop Technologies, Delaware). Following quantity and quality tests based on manufacturer guidelines (NanoDrop 8000, Thermo Scientific Inc., Wilmington, DE, USA), RNA was reverse-transcribed to c-DNA using Qiagen QuantiTect Reverse Transcription Kit (Qiagen; Hilden, Germany) and samples were stored at −20°C for future analyses.

We focused on one of several Hsp70 paralogues in the *Daphnia pulex* reference genome (DappuDraft_302856; [51]). A few primer sets were designed using primer designing and alignment tools taking care to avoid intron–exon boundaries, and phylogenetically variable regions (http://mesquiteproject.org/). We tested the performance of these primers (25 nmol each; Integrated DNA Technologies) in all combinations and picked the pair that resulted in the best amplification of the target gene (hsp70_Forward CATCACTGTTCCTGCCTACTT; hsp70_Reverse AGTGGGCTCGTTGATGATAC) and housekeeping gene (*TATABOX*) (TATA_Forward TCCAAACCGAGGGTCTAGT; TATA_Reverse CATGGGAGCAGGTGTAGATG). Real-time qPCR amplification of the target gene (hsp70) relative to housekeeping (TATA) gene using the ΔΔCt method was employed to quantitate changes in target gene expression due to thermal treatment. qPCR was performed using SYBR green PCR Master Mix (Quanta Biosciences) on the Eppendorf RealPlex2 (Saxony, Deutschland).

### 2.5. Experiment 2: long-term acute thermal shock

The second experiment was conducted for a duration of 72 h. Temperature treatments followed the first experiment (i.e. 28°C, 32°C, 34°C, 35°C and 36°C), but included an additional temperature treatment at 30°C; animals were exposed to sequentially increasing temperatures for 12 h periods. In this experiment, the same nine clones tested in experiment 1 were used along with an additional ancient clone (A3) (the latter was unavailable for experiment 1 due to difficulties synchronizing its reproductive cycle with the other clones).

The pre-experimental protocols for culturing animals followed those described for experiment 1 (see above). The experimental generation for this longer-term heat shock experiment was raised for 7 days at 20°C before exposure to increasing experimental temperature treatments. *Daphnia* were fed 1 mgC l^−1^ HiP *S. acutus* algae every day. For each clone, five replicate jars each containing five animals in 50 ml of COMBO for a total of 50 experimental vessels were placed in a climate-controlled chamber (Percival Scientific, Model I36VL, Perry, IA, USA) during the experimental period. The climate chamber's internal temperature was monitored by both a mercury thermometer and an Onset HOBO U-Series Data Logger® (model: UX 100–001) (Bourne, MA, USA).

Prior to experimentation, daphniids were maintained at room temperature (approx. 22°C) for 24 h. When placed in the chamber, temperature was increased from room temperature over the course of an hour by 1°C increments every 10 min until temperatures reached the initial experimental temperature of 28°C. Thereafter, temperature treatments with a difference of 2°C were increased by approximately 0.3°C every 10 min; temperature treatments with a difference of 1°C were increased by approximately 0.2°C every 10 min.

After 12 h at a given experimental temperature, animals were removed from the chamber and inspected for survivorship. If clutches were released during the experimental period, the number of neonates was recorded and then removed from the jars. Death of an individual was considered, if no movement was observed for 30 s, including a lack of heart beat and lack of filter-feeding. Upon death, animals were removed from the jars. Survivorship was recorded as the proportion of surviving animals per temperature/time treatment per jar. As a control, two replicate jars per clone with five animals each for a total of 20 control vessels were placed at room temperature (approx. 22°C) outside of the chamber. Each 60 ml jar was filled with 50 ml of COMBO [50]; animals were not transferred to new medium to avoid mortality as a result of handling stress. Thus, a total of 50 experimental jars and 20 control vessels were established for this experiment.

### 2.6. Statistical analysis

In addition to tracking survivorship data during both of our experiments, we measured LT50s (i.e. lethal temperature at which 50% of the population had died; [23,52]) to determine whether there were time-period-specific differences in thermal sensitivity. For both experiments, Probit LT50 analyses were conducted. LT50 values were subsequently analysed by a one-way analysis of variance (ANOVA). We tested for significant differences in survivorship by using a general linear mixed model (GLMM) approach for each experiment. Survivorship was our response variable. Temperature and time period were fixed effects; clone and replicate jars were nested within time period and represented random effects. When GLMMs, which test whether survivorship is generally impacted by temperature or time period, were significant, we conducted one-way ANOVAs as a post hoc analysis to elucidate differences in survival among clones from each time period at each temperature treatment. When ANOVAs were significant, we used Tukey post hoc tests (HSD) to determine which time periods differed significantly from each other.

For our heat shock protein analysis, the fold change of the target gene (*hsp70*) was determined relative to the housekeeping gene (*TATABOX*) according to the ΔΔ*C*_T_ method:

$$\text{Fold change}=2^{-\Delta (\Delta C_{T})},$$

where Δ*C*_T_ = *C*_T, target_ − *C*_T, housekeeping_ and Δ(Δ*C*_T_) = Δ*C*_T, treatment_ − Δ*C*_T, control_. A one-way ANOVA was implemented to determine significant differences in log-fold change (*p* ≤ 0.05).

GLMM analyses were conducted using R x64 3.3.1 (nlme package) [53]. All other statistical analyses were conducted using SPSS v. 20 (Armonk, NY, USA).

## 3. Results

### 3.1. Experiment 1: short-term acute thermal shock

**Figure 1.**
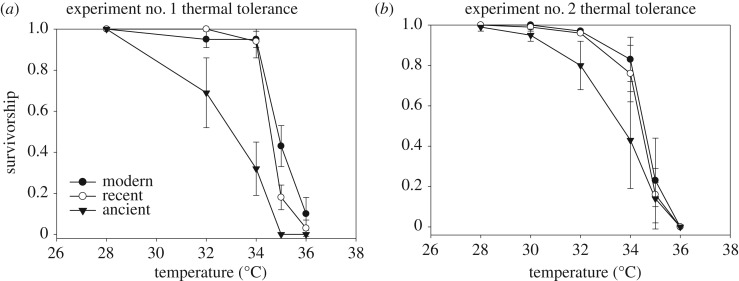
Mean (±1 s.e.) proportion survivorship are shown for the nine *D. pulicaria* clones (i.e. three clones each) from the three distinct (modern, recent, ancient) time periods (i.e. sediment ages) for the first experiment (i.e. short-term acute thermal shock) (*a*) and second experiment (i.e. long-term acute thermal shock) (*b*). Data for clones within time periods were combined.

We hypothesized that clones were adapted to the thermal regimes of their respective time periods; thus, modern clones would have a greater tolerance for heat than recent clones, and even greater tolerance than ancient clones. Results from the GLMM analysis indicated that overall survivorship was not significantly different among modern/recent/ancient time periods (d.f. = 1, *F* = 1.064, *p* = 0.305) across all the temperature treatments. However, analyses ranked time-period survivorships in accordance with our hypothesis, such that modern > recent > ancient. Separate post hoc ANOVA tests were conducted to elucidate differences among time periods at specific temperature treatments for further exploration of our hypothesis. These results indicated that survivorship was significantly different across time periods at 32°C (d.f. = 2, *F* = 8.137, *p* = 0.002), 34°C (d.f. = 2, *F* = 60.951, *p* < 0.0001) and 35°C (d.f. = 2, *F* = 16.624, *p* < 0.0001) (table 1 and figure 1*a*). Furthermore, Tukey HSD tests revealed that both the modern and recent time periods differed from ancient at 32°C (*p* = 0.013 and 0.003, respectively), at 34°C (both *p* < 0.001), but did not differ from each other at these two temperatures. At 35°C, all time periods were significantly different from one another. The LT50 for the modern time period was estimated at 34.87°C, recent at 34.59°C and ancient at 32.96°C. LT50s across time periods differed significantly (d.f. = 2, *F* = 18.321, *p* < 0.0001) (electronic supplementary material, figure S1a) and were ranked in accordance with our hypothesis, such that modern > recent > ancient. Of the 45 individuals in control (20°C) jars, two deaths were observed during the experimental period (one M2, and one R3).

**Table 1.** Post hoc ANOVA results of survivorship across time periods (top) and clones (bottom) for the first experiment (i.e. short-term acute thermal shock). The range of temperature treatments is given (left-hand side). Significant test results are in bold. Note: there are no values for 28°C as there was 0% mortality after exposure to that temperature treatment.

temp (°C)	d.f.	*F*	Sig.
time period			
32	2	8.137	**0**.**002**
34	2	60.951	**0**.**000**
35	2	16.624	**0**.**000**
36	2	2.440	0.108
clones			
32	8	3.591	**0**.**012**
34	8	21.212	**0**.**000**
35	8	4.286	**0**.**005**
36	8	1.541	0.212

**Figure 2.**
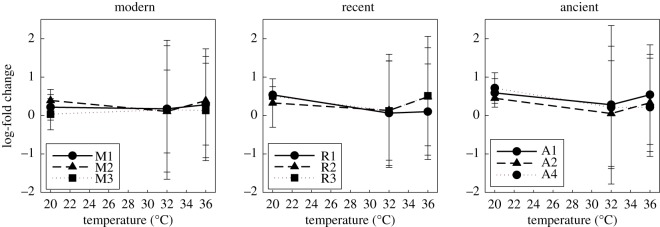
Log-fold change of hsp70 gene expression profiles from the first experiment (short-term acute thermal shock) for the nine *D. pulicaria* clones (*n* = 3) from the three distinct time periods (modern, recent and ancient). All clones within a time period were isolated from the same sediment layer. Time periods are designated as modern (M), recent (R) or ancient (A) (see Material and methods for details).

Results did indicate significant differences in log-fold change of hsp70 gene expression across time periods at the three temperatures. Clones differed significantly in hsp gene expression at 20°C (d.f. = 8, *F* = 6.355, *p* = 0.001) and 32°C (d.f. = 8, *F* = 5.771, *p* = 0.001), but Tukey HSD analyses indicated no significant differences across and within time periods (table 2 and figure 2). Thus, it appears there is clonal variation, but it is not consistent with our hypothesis of time-period-specific (i.e. sediment layer) differences in hsp gene expression. Moreover, a Pearson correlation was conducted at 32°C and 36°C (i.e. the two temperatures that were common to the survivorship and hsp70 experiments) to investigate the relationship between survivorship and gene expression. Analyses revealed that log-fold change was unrelated to mortality across clones at 32°C (*p* = 0.310) or 36°C (*p* = 0.892).

**Table 2.** ANOVA results across time periods (top) and clones (bottom) of hsp70 log-fold change for the first experiment (i.e. short-term acute thermal shock) for three temperatures (20°, 32° and 36°C). Significant test results are in bold.

temp (°C)	d.f.	*F*	Sig.
time period			
20	2	0.608	0.553
32	2	2.003	0.157
36	2	0.250	0.781
clones			
20	8	6.355	**0.001**
32	8	5.771	**0.001**
36	8	0.700	0.688

### 3.2. Experiment 2: long-term acute thermal shock

**Figure 3.**
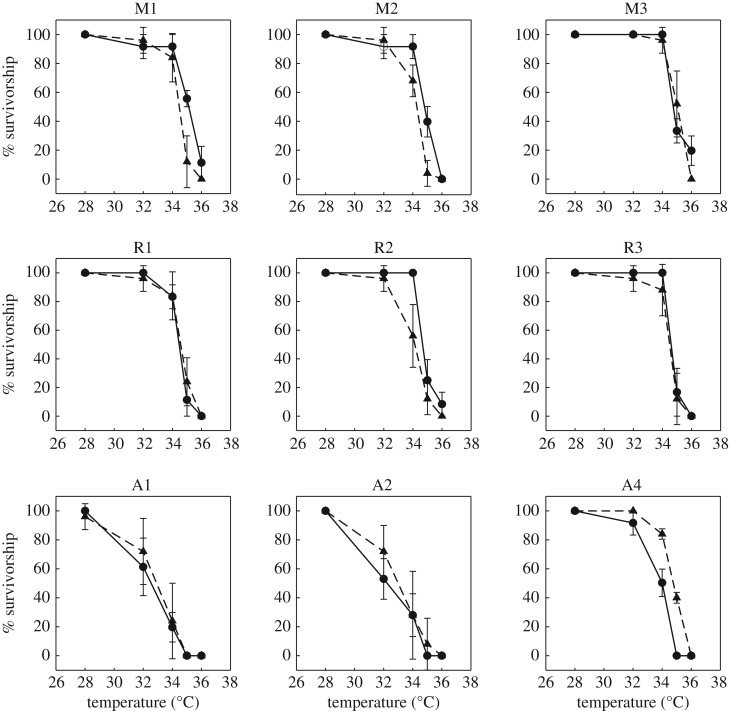
Mean (±1 s.e.) clone-specific survivorship comparisons between the first (i.e. short-term acute thermal shock, circles) experiment and the second (i.e. long-term acute thermal shock, triangles) experiment for the nine *D. pulicaria* clones that were tested in both experiments. (Note: the 10th clone, A3, was only tested in the second experiment and is not shown here; see electronic supplementary material). Clonal designations are given at the top of each plot. The top row represents the clones from the modern (M) time period, the middle row shows the clones from the recent (R) time period, and the bottom row indicates the clones from the ancient (A) time period. All clones within a time period (e.g. M1, M2, M3) were isolated from the same sediment layer (see Material and methods section for details).

Results from the GLMM indicated that overall survivorship was significantly different among clones from the modern/recent/ancient time periods (d.f. = 1, *F* = 8.254, *p* < 0.0044) for the entire temperature series. However, analyses did not rank time-period survivorship in accordance with our hypothesis for each temperature treatment; it appears that there was variability in survivorship based on time period. Separate post hoc ANOVA tests were conducted to elucidate significant differences among time periods at specific temperature treatments for further exploration of our hypothesis. These results indicated survivorship was significantly different across time periods at 32°C (d.f. = 2, *F* = 7.608, *p* = 0.001) and 34°C (d.f. = 2, *F* = 4.711, *p* = 0.014) (table 3 and figure 1*b*). Further, Tukey HSD tests revealed that the modern and ancient time periods differed significantly at these two temperature treatments (*p* = 0.003 and 0.022, respectively); recent and ancient also differed significantly at these temperature treatments (*p* = 0.009 and 0.053, respectively), but modern and recent did not significantly differ from each other for either of these temperature treatments. LT50 for the modern time period was estimated at 34.38°C, recent at 34.14°C and ancient at 33.31°C. These values were significantly different from one another (d.f. = 2, *F* = 3.283, *p* = 0.042). Tukey HSD tests revealed that LT50 of the modern time period was significantly different from the ancient time period (*p* = 0.043); neither were significantly different from recent (electronic supplementary material, figure S1b). Further, observations of independent clones within each time period lend support to these findings (figure 3). No mortality was observed in the control (approx. 22°C) jars during the course of this experiment.

**Table 3.** Post hoc ANOVA results of survivorship across time periods (top) and clones (bottom) for the second experiment (i.e. long-term acute thermal shock). The range of temperature treatments is given (left-hand side). Significant test results are in bold. Note: there are no values for 36°C as there was 100% mortality after exposure to this final temperature treatment.

temp (°C)	d.f.	*F*	sig.
time period			
28	2	0.742	0.482
30	2	2.994	0.060
32	2	7.608	**0.001**
34	2	4.711	**0.014**
35	2	0.252	0.778
clones			
28	9	1.000	0.456
30	9	1.422	0.211
32	9	4.217	**0.001**
34	9	5.132	**0.000**
35	9	3.269	**0.005**

## 4. Discussion

Overall, results from our two acute-thermal tolerance experiments lend support to our original hypothesis of differential thermal tolerance among genotypes from different time periods. Survivorship differences were significant for the second thermal shock experiment, but not for the first (i.e. shorter-duration) experiment, although trends in survivorship for this experiment offer support for our hypothesis. Moreover, post hoc tests on clonal survivorship from the three time periods in this experiment responded in a predictive manner, revealing significant differences at three of the temperature treatments. Specifically, modern clones had higher survivorship than the recent clones, while ancient clones had the lowest survivorship rates as temperature increased in both experiments.

LT50 estimates followed the predictive trend, such that modern clones exhibited higher temperature tolerance than recent clones, and recent were greater than ancient. Post hoc testing on survivorship revealed that the modern and ancient time periods were significantly different, as were LT50s in both experiments. Lastly, although the data do not support our hypothesis that hsp70 would be differentially expressed between time periods, our results demonstrate an upregulation of hsp70 in response to temperature. Although it was not in accordance with our hypothesis, we did detect increased production of this protein in response to temperature. These clones are clearly responding to thermal stress, but differential production of this protein may not be the mechanism by which clones are coping with stress.

It is evident from this study that modern and recent clones have adapted to warmer temperatures and are able to tolerate acute thermal stress better than ancient clones. Modern and recent clones had similar responses, which did not significantly differ overall. Hsp70 production did not reveal significant differences among the clones from the three time periods, but indicate a response to handling and coping with immediate heat stress. Taken together, it is highly plausible that temporal subpopulations of *D. pulicaria* from SC Lake are locally adapted to the temperatures of their respective time periods. Further experimentation is required to provide unequivocal support for our conclusions.

We do recognize a limitation of our study, namely the low number of clonal representatives from each time period. Hatching success of ancient clones was very low (i.e. approx. 0.03–1.0%; [43]) and we aimed to maintain approximately equal numbers of clones per time period in this experiment (i.e. three modern, three recent, three/four ancient design). Despite this limitation, we do not believe this impacted the overall results of our experiments, given that these clones are genetic representatives of their time periods [37] and provide us with an idea of the microevolutionary potential of past populations in the face of changing environmental variables.

Interestingly, LT50 estimates were concordant in both experiments, revealing similar trends and predictive patterns for the three time periods. Despite differences in length of exposure to temperature treatments (i.e. 30 min intervals in the first experiment, 12 h intervals in the second experiment), it is not surprising that they are so similar, given that many organisms have both shorter-term and longer-term response mechanisms to deal with thermal stress, which include heat hardening, acclimation, acclimatization and (ultimately) adaptation [54]. The differences in exposure time, as well as acclimation period between the two experiments that lead to differences in thermal response, most likely reflect such differential thermal response mechanisms (i.e. fast-response versus slower-response), as outlined by Huey & Bennett [54]. Thus, the overall patterns among clones from different time periods did not differ between these two experiments (figure 3), thus strengthening our findings.

Other studies have illustrated comparable results for local adaptation to thermal regimes. Tobler *et al*. [55] reported similar results using *Drosophila melanogaster* as their model organism. *Drosophila* base populations were established in either bimodal cyclic ‘hot’ (18–28°C) or ‘cold’ (10–20°C) conditions for evaluation of fitness and tolerance traits after 40 generations of exposure to either hot or cold thermal stress; populations optimally adapted to their respective thermal environments. Therefore, descendants of hot-based populations were more tolerant of hot thermal stress conditions, and only hot populations were found to be significantly more fit under their respective thermal regimes [55]. In another recent study, Geerts *et al*. [25] also employed a resurrection ecology approach and found evidence for local adaptation to changes in thermal regimes. Twelve clones of *Daphnia magna* were hatched from a historic lake sediment core (1955–1965) and 12 clones were hatched from a recent core (1995–2005), both extracted from Felbrigg Hall Lake, UK. Clones were exposed to increasing temperatures; recent clones were found to have significantly higher critical thermal maxima values when compared with historic clones. Moreover, in a separate experiment, 150 clones of *D. magna* were exposed to either ambient or ambient + 4°C treatments in mesocosm tanks. After 2 years, cores were extracted and 20 clones from ephippial hatchlings were established. Hatched clones were subsequently exposed to rising temperatures; clones from heated mesocosms had 3.6°C higher critical thermal maxima on average than non-heated mesocosm clones. Results from both experiments outlined here suggest that natural populations of *Daphnia* have the ability to evolve heat tolerance, and have the evolutionary potential to genetically respond to acute changes in the thermal regimes of their immediate habitat [25]. Our results lend support to these earlier observations.

In a previous study conducted by Yousey & Weider (personal observation), life histories of these 10 experimental clones were not found to vary between time periods, despite exposure to temperature treatments mimicking environments of the three time periods. Rather, plasticity among clones was detected and, thus, it would seem that differential life histories would not affect the results of this current study. Other previous studies that used a number of these same clones investigated the effects of eutrophication on the evolutionary trajectory of this population [37,41,43]. Frisch *et al*. [37] examined nine of the 10 clones used in this study, and found that these clones exhibited physiologically distinct phenotypes for C : P processing, which may impact competitive interactions among clones [43]. Further, Roy Chowdhury *et al*. [41] found distinct differences between modern and ancient clones in gene expression patterns, when exposed to high-P and low-P environments. Taken collectively, these studies provide further validation of the impacts of increased phosphorus supply and its impact on the underlying population genetic structure over time.

Altogether, it is apparent that these physiological traits can impact clonal survivorship over time; however, the magnitude of this impact in a changing climate scenario still needs to be evaluated. Collectively, the research conducted on these clones points to multifarious selection and adaptation to a number of anthropogenic stressors. Nutrient supply will continue to affect this species under future climate change scenarios, as will temperature [37,41,43]; their interactive effects will require further investigation.

Thermal acclimation experimentation may have produced results different from those reported in this study. Several studies have examined the thermal tolerance of a number of organisms by acclimatizing a subset of experimental animals to multiple temperatures followed by exposure to rising temperature treatments [23,52,56–58]. Using the acclimation approach followed by exposure to thermal shock, these studies revealed that as acclimation temperatures increased, so do upper thermal limits and survivorship rates as animals are exposed to rising temperatures [52,56–58]. Although an acclimation approach was not feasible in this study due to an insufficient quantity of animals to adequately compare thermal tolerance ranges, it would be interesting to explore thermal tolerance with our clones in this manner. However, acclimation is not necessarily essential to test our hypothesis. Our objective was to produce evidence for temporal local adaptation to temperature rather than an exploration of differences between thermal tolerance ranges, although the latter is indeed important.

Although expression of hsp70 was sensitive to temperature, expression was similar between the more tolerant modern and the susceptible ancient genotypes. This is not too surprising as we tested a single set of hsp70 primers, which amplifies one hsp70 isoform in the genome. There are several isoforms of the hsp70 protein present in organisms, which have been demonstrated to have a wide range of responses to stress [26,59]. Amplification of specific hsp70 isoforms would potentially enhance our understanding of the heat tolerance results and LT50 estimates. Interestingly, previous work by Mikulski *et al*. [27] has shown that in response to thermal stress, a single clone of *D. magna* exhibited differential expression of isoforms of hsp70, with upregulation of the 73 and 74 kDa proteins, and inhibition of the 79 kDa protein. Whether such a response varies among genotypes/clones of *Daphnia* remains an open question. Although we did not find a significant difference in protein expression, there is clearly a response to thermal stress. It is possible that there could be variability in the expression of other isoforms of hsp70, and warrants further research.

In a recent study, Mikulski *et al*. [60] explored the effect of diel vertical migration (DVM) behaviour and thermal stress on *Daphnia* growth rates and hsp70 expression. The study used clones from four different types of aquatic habitats, including shallow ponds and thermally polluted lakes in northern Europe, and investigated the ability of these genotypes to cope with short-term and long-term warming. Results revealed that rapid temperature changes as a result of DVM and rapidly increasing temperature had a negative impact on juvenile growth rates. They also found that a more rapid change in temperature had a greater impact on energetic costs than slower changes. Lastly, the study revealed clonal effects on the ability to cope with thermal stress and found lower levels of hsp70 expression in the presence of rapid temperature change. While we did not quantify DVM behaviour, clonal differences (in terms of time period) and higher rates of survivorship in the long-term acute thermal shock experiment are similar to observations by Mikulski *et al*. [60]. Moreover, this study found that the rate of experimental temperature change impacts hsp70 expression, suggesting that such effects may become apparent with a different rate of temperature change than the one we employed for the short-term acute thermal shock experiment that did not detect any differences in hsp70 expression.

Climate change predictions reported by the IPCC [1] suggest that under scenarios of increased temperature and precipitation (and with the interaction of other environmental factors), dissolved organic carbon (DOC) will increase in lakes, where our model organism resides. Along with increasing ambient temperatures, an increase in DOC will affect surface water temperatures in aquatic systems by increasing the absorbance of infrared radiation, thus resulting in higher surface water temperatures. This will cause a greater temperature differential between surface and bottom waters, which will result in more strongly (thermally) stratified systems. Such changes will have dramatic impacts on ecosystem processes such has predator–prey interactions (i.e. DVM—of zooplankters like *Daphnia*; [16]), ultraviolet radiation (UVR) impacts [61] as well as a host of other impacts (e.g. physiological, behavioural) on keystone organisms like *Daphnia* [16].

In addition to rising water and air temperatures, we also expect to observe increased lake nutrient content [1]. However, the rate at which lakes warm does not always correlate with air temperature. Rather, due to spatial heterogeneity, we predict variability in climate change effects across lakes [9]. Nonetheless, it is clear that under such climate change scenarios, *Daphnia* would be greatly impacted as this genus is sensitive to lake nutrient contents and elevated temperatures [11,37,41,62]. As a keystone species, the broader impact and consequences of warming on this organism could potentially lead to detrimental consequences across multiple trophic levels [16–18].

In conclusion, our findings suggest that rising temperatures have played a role in shaping the microevolutionary history of the *Daphnia* (sub)populations in South Center (SC) Lake, over time. For lakes like SC that are experiencing eutrophication (via increased nutrient loading—primarily phosphorus loading; [37]) and climate change, such multiple stressors may lead to shifts in community composition that can impact overall levels of biodiversity [1]. Thus, it is important to consider the interactive effects of multiple stressors when examining the response of species to climate change beyond warming itself. Investigation of these interactions is a critical, primary focus of future research [5]. Specifically, the interaction between temperature and phosphorus is warranted, as phosphorus supply (as noted above) has been observed to impact SC subpopulations of *Daphnia* over time [37,41,43]. Resurrection ecology approaches, where applicable, are useful in understanding long-term consequences of thermal stress [25] and its interactive effects with other anthropogenic stressors on the health and microevolutionary responses of natural populations.

## Supplementary Material

Supplemental Figure 1

## Supplementary Material

Supplemental Figure 2

## Data accessibility

Our data are deposited at Dryad: https://dx.doi.org/10.5061/dryad.f30r1 [63].
