# Supplementary material for: Resurrected ‘ancient’ Daphnia genotypes show reduced thermal stress tolerance compared to modern descendants
Source: R Soc Open Sci. 2018 Mar 21;5(3):172193. doi: 10.1098/rsos.172193 (PMC5882736; doi:10.1098/rsos.172193)
Supplement: Supplemental Figure 1 [file rsos172193supp1.pdf]

Supplemental Figure 1. LT50s (a & b) are shown for the 9 *Daphnia pulicaria* clones (i.e., three clones each) from the three distinct (modern, recent, ancient) time periods (i.e., sediment ages) for the first experiment (i.e., short-term acute thermal shock) (a) and second experiment (i.e. long-term acute thermal shock) (b). Y-axis indicates (mortality) probit values.

a)

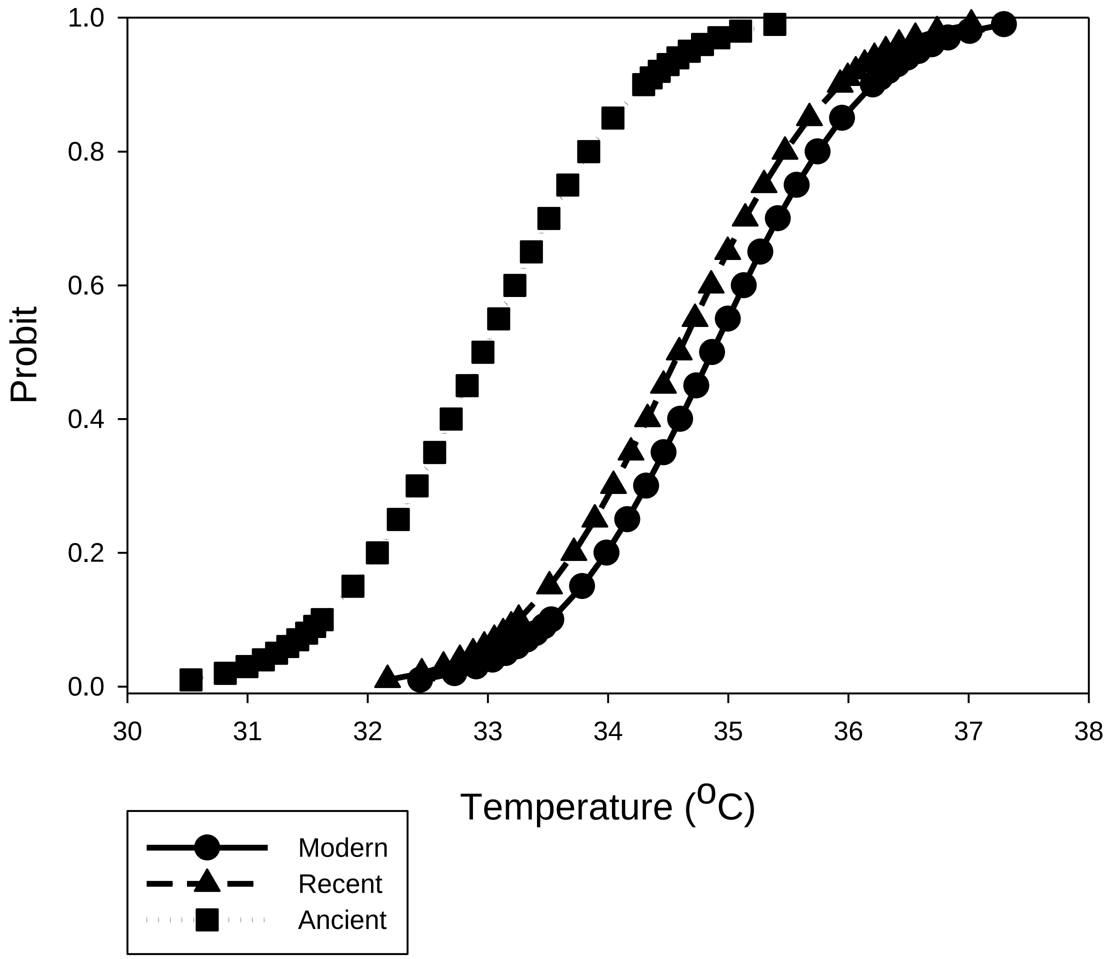

b)

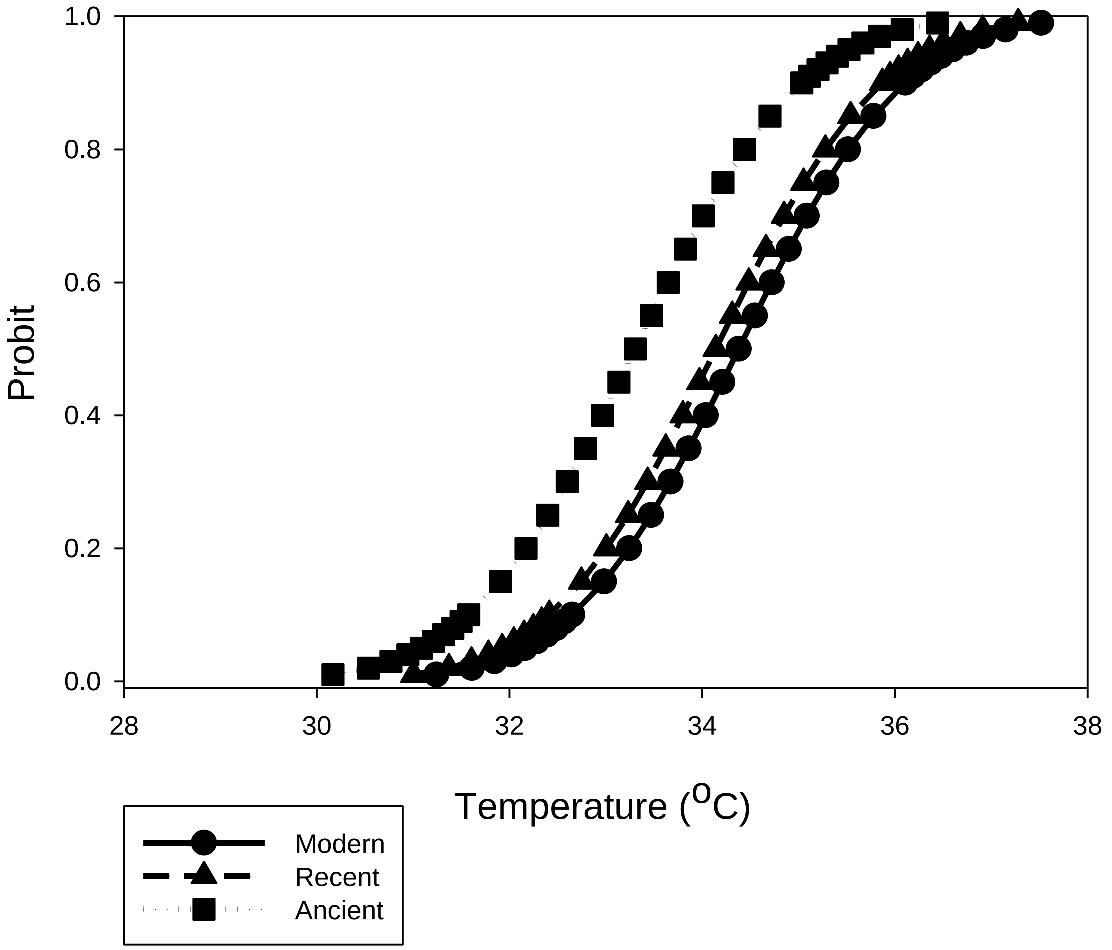

1.0
0.8
0.6
0.4
0.2
0.0
Probit
28
30
32
34
36
38
Temperature (^{o}C)
Modern
Recent
Ancient
