# Supplementary material for: Resurrected ‘ancient’ Daphnia genotypes show reduced thermal stress tolerance compared to modern descendants
Source: R Soc Open Sci. 2018 Mar 21;5(3):172193. doi: 10.1098/rsos.172193 (PMC5882736; doi:10.1098/rsos.172193)
Supplement: Supplemental Figure 2 [file rsos172193supp2.pdf]

Supplemental Figure 2. Mean (± 1 S.E.) clone-specific survivorship for clone A3, which was only tested in the second (i.e., long-term acute thermal shock) experiment.

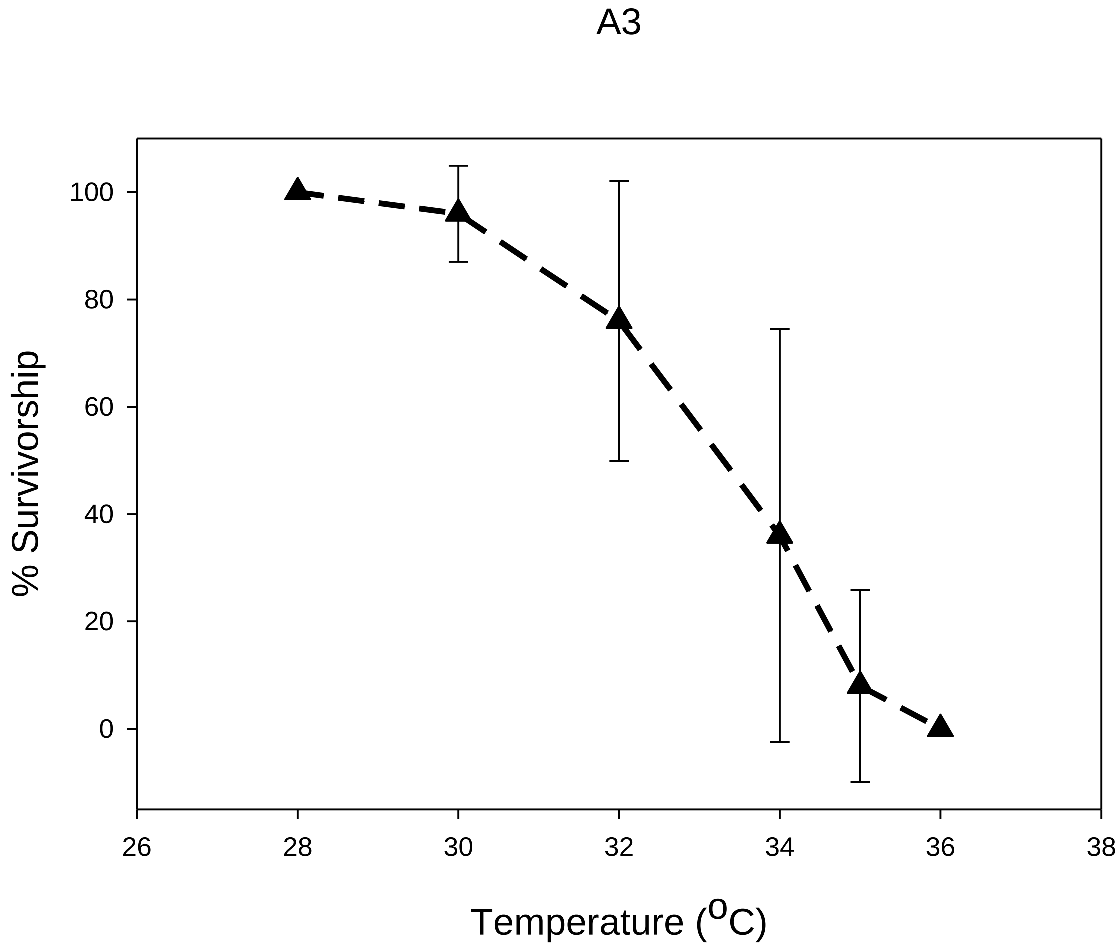

Correspondence: Aime’e Yousey. Department of Biology, University of Oklahoma. 730 Van Vleet Oval, E Norman, OK, USA 73019. Email: ayousey@ou.edu.
